# De Novo Mutation in KMT2C Manifesting as Kleefstra Syndrome 2: Case Report and Literature Review

**DOI:** 10.3390/pediatric14010019

**Published:** 2022-03-11

**Authors:** Maria Anna Siano, Ilaria De Maggio, Roberta Petillo, Dario Cocciadiferro, Emanuele Agolini, Massimo Majolo, Antonio Novelli, Matteo Della Monica, Carmelo Piscopo

**Affiliations:** 1 Department of Medicine, Surgery and Dentistry “Scuola Medica Salernitana”, Postgraduate School of Pediatrics, University of Salerno, 84084 Salerno, Italy; mariaannasiano@gmail.com; 2 Medical and Laboratory Genetics Unit, A.O.R.N. ‘’Antonio Cardarelli’’, 80131 Naples, Italy; ilaria.demaggio@aocardarelli.it (I.D.M.); roberta.petillo@aocardarelli.it (R.P.); matteo.dellamonica@aocardarelli.it (M.D.M.); 3 Laboratory of Medical Genetics, Bambino Gesù Children Hospital, 00165 Rome, Italy; dario.cocciadiferro@opbg.net (D.C.); emanuele.agolini@opbg.net (E.A.); antonio.novelli@opbg.net (A.N.); 4 Hospital Directorate, National Hospital A.O.R.N. ‘’Antonio Cardarelli’’, 80131 Naples, Italy; massimo.majolo@aocardarelli.it

**Keywords:** KMT2C, Kleefstra syndrome 2, intellectual disability

## Abstract

Diagnosis of pediatric intellectual disability (ID) can be difficult because it is due to a vast number of established and novel causes. Here, we described a full-term female infant affected by Kleefstra syndrome-2 presenting with neurodevelopmental disorder, a history of hypotonia and minor face anomalies. A systematic literature review was also performed. The patient was a 6-year-old Caucasian female. In the family history there was no intellectual disability or genetic conditions. Auxological parameters at birth were adequate for gestational age. Clinical evaluation at 6 months revealed hypotonia and, successively, delay in the acquisition of the stages of psychomotor development. Auditory, visual, somatosensory, and motor-evoked potentials were normal. A brain MRI, performed at 9 months, showed minimal gliotic changes in bilateral occipital periventricular white matter. Neuropsychiatric control, performed at 5 years, established a definitive diagnosis of childhood autism and developmental delay. Molecular analysis of the exome revealed a novel KMT2C missense variant: c.9244C > T (p.Pro3082Ser) at a heterozygous state, giving her a diagnosis of Kleefstra syndrome 2. Parents did not show the variant. Literature review (four retrieved eligible studies, 10 patients) showed that all individuals had mild, moderate, or severe ID; language and motor delay; and autism. Short stature, microcephaly, childhood hypotonia and plagiocephaly were also present. Conclusion. Kleefstra syndrome 2 is a difficult diagnosis of a rare condition with a high clinical phenotypic heterogeneity. This study suggests that it must be taken in account in the work-up of an orphan diagnosis of intellectual disability and/or autism spectrum disorder.

## 1. Introduction

Intellectual disability (ID) disorder is a composite group of disorders characterized by significantly damaged intellectual functioning and adaptive behaviors deficiency [1]. About 1–3% of the western population are affected and characterized by genotypes and phenotypes which are highly heterogeneous [2]. The etiological factors of ID are very varied and in many children the cause of ID is still unknown [2]. Genetics plays a relevant role in its development; indeed, advanced sequencing methods have identified mutated genes in intellectual disability, autism, and other disorders [3]. For instance, a recent study stressed the importance of rare heterozygous de novo mutations as a cause of undiagnosed developmental disorders [4]. Developmental disorders caused by de novo mutations have an average prevalence of 1 in 213 to 1 in 448 births in relation to parental age and globally account for about 400,000 affected children born annually [4].

In the last few years, use of diagnostic whole exome sequencing for undetermined neurodevelopmental disorders identified de novo KMT2C mutations.

In this paper we report a novel patient with ID, autism, and minor facial dysmorphisms displaying a missense heterozygous mutation in KMT2C, compatible with diagnosis of Kleefstra syndrome 2 (KLEFS2). A systematic literature review of other cases of KLEFS2 was also performed.

## 2. Methods

### 2.1. Literature Review

We used Preferred Reporting Items for Systematic Reviews and Meta-Analyses (PRISMA) flowchart of the literature search results. We consulted the PubMed, Scopus and Choocrane Library academic medical databases. Our search strategy was based on the terms “KMT2C” and “Kleefstra syndrome 2”. Systematic search of the literature databases was performed with no language restrictions, and it considered publications from January 2000 to February 2021. To be suitable for inclusion, studies had to describe a case of KLEFS2. Three authors took part. They had separately selected articles and took a stepwise approach, first by regarding the title, then by reviewing the abstract, and, as a third step, by revising the full text, where appropriate (Figure S1). Finally, four studies were selected. All the details about the articles included and excluded has reported in Supplementary Figure S1.

### 2.2. Material and Methods WES

Library elaboration and whole exome capture were performed by using the Twist Human Core Exome Kit (Twist Bioscience) according to the manufacture’s protocol. The sequencing was carried out on the Illumina NovaSeq 6000 platform. The BaseSpace pipeline (Illumina) and the TGex software (LifeMap Sciences) were employed for the calling- and annotating variants, respectively. Sequencing data were aligned to the hg19 human reference genome. A minimum depth coverage of 30× was considered eligible for analysis, according to the guidelines of the American College of Medical Genetics and Genomics. Variants were analyzed for coverage and Qscore (minimum threshold of 30), and visualized by the Integrative Genome Viewer (IGV).

## 3. Case Presentation

**Figure 1:**
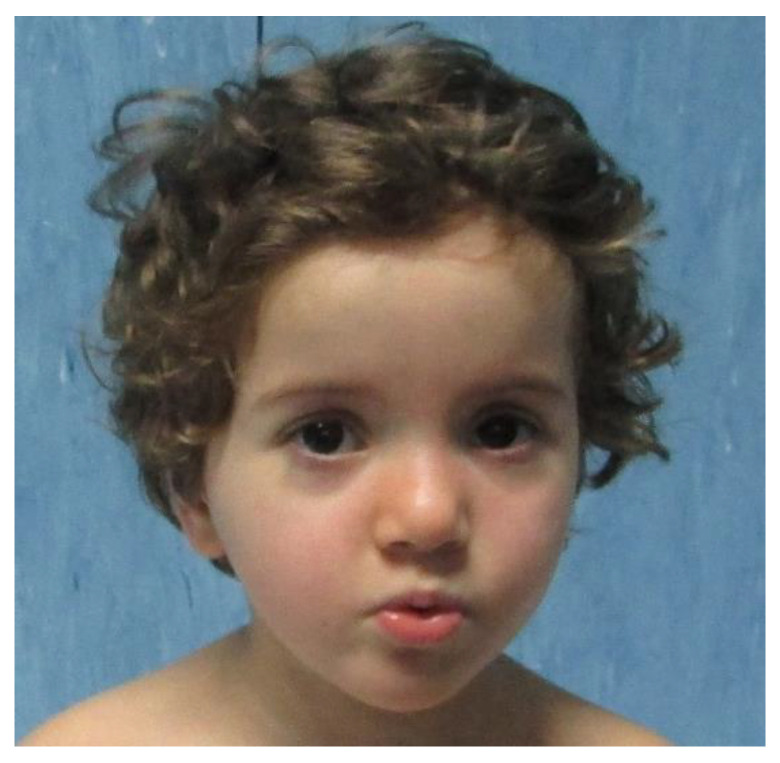
Clinical photographs of our patient at first clinical examination.

The proband was a 6-year-old Caucasian female referred to our center for neurodevelopmental disorder, history of hypotonia, and minor face anomalies. The family history was negative for intellectual disability or genetic conditions. She was born to non-consanguineous parents at 39 weeks of gestation after a normally conducted pregnancy until the seventh month when there was an onset preeclampsia, and a caesarian section was performed. She was deemed adequate for her gestational age: birth weight was 3460 g (75th percentile), length 50 cm (50–75th percentile) and head circumference 35 cm (75–90th percentile). The Apgar score at 1 and 5 min was 8/9. Clinical evaluation at 6 months revealed hypotonia and, successively, delay in the acquisition of the stages of psychomotor development. Auditory, visual, somatosensory, and motor evoked potentials were normal. A brain MRI, performed at 9 months, showed minimal gliotic changes in bilateral occipital periventricular white matter. Control MRI, performed a year later, showed improvement of the alteration of the posterior periventricular signal in relation to myelination phenomena, low-signal stria in the supratrigonal area from a likely vascular element, and modest ectasia of the regional perivascular spaces. The childhood neurological and neuropsychiatric visits ended with psychomotor delay and general developmental disorder characterized by the absence of language, a tendency to isolation, with little interest and inconsistent visual engagement. Autistic traits characterized by poor participation, repetitive behaviors, unusual interest in objects, symptoms that resemble attention deficit hyperactivity disorder and mild macrocrania, motor stereotypies, and broad-based walking were also found. Cardiac evaluation performed at 2 and 6 years revealed patent foramen ovale and abdominal ultrasound was normal. Neuropsychiatric control, performed at 5 years, established a definitive diagnosis of childhood autism and developmental delay. Peripheral blood karyotype analysis was 46, XX (female, normal). To exclude the presence of small cytogenetic anomaly, we performed high resolution array-CGH, which was revealed to be very small at 2p16.3 microdeletion (87 kb) with maternal transmission, involving part of the first intron, part of the second intron, and the second exon of the NRXN1 gene, without a clear pathogenetic role considering size, transmission with a large presence in other family healthy controls (mother, 2 of 3 brothers of the mother and 2 of 5 maternal cousins of proband), and incomplete penetrance of the NRXN1 deletion syndrome. No mutations were found sequencing the NRXN1 and ZEB2 genes. To rule out Angelman syndrome, methylation and MLPA tests of chromosome 15q11–13 were also performed and were normal. Whole exome sequencing of the patient and her parents was performed on genomic DNA obtained from peripheral blood leucocytes, identifying the de novo c.9244C > T; p.Pro3082Ser missense variant. According to the Combined Annotation Dependent Depletion score (CADD score 22) and according to the American College of Medical Genetics score (PS2, PM2, PP3) [5] this variant can be considered as likely pathogenic. CADD has become one of the most widely used tools to assess human genetic variation and values > 20 indicate that a variant is more likely to have deleterious effects. The final diagnosis for the patient was Kleefstra syndrome 2. The last examination at 6 years and 9 months still revealed the absence of both language and independent walking. Irritability, hyperactivity, self- and hetero-harm were observed. The child showed macrocephalic facies, broad and rounded forehead, hypertelorism, nose with a saddle bridge and bulbous tip, slight telelia, ligamentous hyperlaxity, and a café au lait spot on the left thigh (Figure 1).

## 4. Discussion

KMT2C mutations cause Kleefstra syndrome 2 (OMIM 617768), a neurodevelopmental disorder characterized by delayed psychomotor development, intellectual disability, and mild dysmorphic features. Inheritance is autosomal dominant.

KMT2C maps to chromosome 7q36 and is highly expressed in the cerebellum of the developing and adult human brain [6,7]. This gene encodes histone-lysine N-methyltransferase 2C, an enzyme that monomethylates lysine 4 of histone H3 (H3K4me1) thus leading to chromatin structure changes that cause transcriptional activation which regulates gene transcription [8]. Genes encoding regulators of H3K4 methylation are known to be among the strongest genetic risk factors for intellectual disability and autism spectrum disorders, having been associated with monogenic forms of neurodevelopmental disease [8]. It is interesting to note that one reported individual has hypoplasia of the cerebellar vermis [9], which is absent in the others.

KMT2C gene constitutes the core of nuclear regulatory structures together with the KMT2D gene, called the KMT2C/D COMPASS complex (complex of proteins associated with Set1). Over the past two decades, mutations in five core genes of the COMPASS complex have been associated with three known human syndromes: Kabuki syndrome type 1 and 2 (KMT2D, KDM6A), Rubinstein-Taybi syndrome type 1 and 2 (CBP, EP300), and type 2 Kleefstra syndrome (KMT2C). Phenotypic similarities and differences can be found between the members of this new family of diseases [10].

Recently, a KMT2C gene mutation has been identified as a cause of familial non-syndromic primary teeth retention, suggesting that KMT2C is also involved in the physiological eruption of permanent teeth, without other phenotypic abnormalities [11]. However, there is no history of this disorder both in our case and in the other description patients.

The novel variant found is not reported in the public databases of mutations/polymorphisms. According to the criteria of the American College of Medical Genetics and Genomics (ACMG) for the interpretation of variants, this can be classified as likely pathogenic. The identification of this novel mutation, with the review of previously published cases, allows us to better define the clinical phenotype related to KMT2C mutations (Table 1). As shown in Table 1, all reported cases had ID, ranging from mild to severe, developmental delay, and autism or autistic spectrum disorders such as pervasive developmental disorder (PDD). Other recurrent clinical features were short stature (5/11), microcephaly (5/11), childhood hypotonia (4/11), and plagiocephaly (3/11). Kleefstra-like facial dysmorphisms are variables as shown in Table 2.

## 5. Conclusions

Kleefstra syndrome 2 is a rare condition, and its diagnosis is arduous principally due to high clinical phenotypic heterogeneity. With this report we suggest considering Kleefstra syndrome 2 as an etiological cause of autism with unknown pathogenesis and report the novel c.9244C > T (p.Pro3082Ser) variant as causative of this condition. A specific diagnosis has a critical role for management of the disease, prediction of possible problems, prognosis, and prenatal or preimplantation diagnosis, and may participate in the advance of eventual future therapies. Finally, this report summarizes previously reported clinical manifestations of Kleefstra syndrome 2, comparing them with these present in our patient and provide a guidance for better counseling of future Kleefstra syndrome 2 patients and their families.

## Figures and Tables

**Table 1:** Clinical and genetic characteristics of affected individuals with KMT2C mutation.

Reference	Total Cases	Sex/Age	KMT2C Gene Mutations Additional De Novo Mutation				Growth		Development			Neurological	Other	MRI
			Chromosome Position (Hg19)	cDNA Change	Amino Acid Change	Deletion	H. (SD)	W. (SD)	H.C. (SD)	I.D.	L.D. M.D			
Kleefstra et al. [12] (2012)	1	F/ 15 years	g.151891591G > A	c.4441 C > T	p.(Arg 1481*)	-	−2.5	0	−2	Moderate IQ 35	Yes	hyperactivity, aggressiveness. Hypotonia	-	N.R.
Koemans et al. [13] (2017)	5	M/ 29 years	g.151880108del	c.5216 del	p.(Pro 1739Leufs*2)	-	−1.7	+0.6	−0.5	Moderate	Yes	Autistic-traits. Epilepsy	PKU, RRI	N.R.
		M/ 31 years	g.151874988G > C	c.7550C > G	p.(Ser 2517*)	-	−0.5	−1.5	−0.5	Mild	Yes	Autism	Strabismus, cryptorchidism	N.R.
		M/ 15 years	g.151947983T > A	c.1690 A > T	p.(Lys 564*)	-	−2	+1.7	−0.6	Moderate IQ 50	Yes	PDD-NOS, ADHD Hypotonia	Bifid uvula, hypospadia, bilateral inguinal hernia	Normal
		F/ 7 years	g.151859847_151859850 del	c.10812_ 10815 del	p.(Lys 3605Glufs*24)	-	−3	−1.5	−2.25	Mild IQ 63	Yes	Autism, sleeping disorder	RRI, dry skin, hoarse voice	Normal
		F/ 10 years	-	-	-	7q36.1 (151,858,920– 152,062,163) ×1	N.R.	−2.5	−2	Severe	Yes	Automutilation Hypotonia. Epilepsy	-	Non-progressive enlarged extracerebral space
Faundes et al. [9] (2018)	3	F/ 17 years	7:151,884,849	c.4744 G > T	p.(Gly 1582*)	-	−2.1	−2.7	−2.42	Severe	N.R	Elective mutism	Delayed puberty	N.R.
		F/ 4 years	7:151,873,688- 151,873,689	c.8849_ 8850 delAT	p.(His 2950 Argfs*17)	-	−2	−2	−1.97	Severe	-/ Mild	-	-	Hydrocephalus hypoplasia of cerebellar vermis
		F/ 5 years	7:151,836,279	c.14526dupG	p.(Pro 4843Alafs*12)	-	0.4	0.18	−1	Severe	N.R.	Autistic traits, developmental regression, insensitivity to pain and abnormal gait	Constipation	N.R.
Schoch et al. [14] (2020)	1	F/ 6 years	-	-	-	7q36.1 (151,839,151–151,965,981) ×1	N.R	N.R	+2	Mild IQ 81	Yes	N.R.	Torticollis	N.R.
Our case	1	F/ 6 years	-	c.9294 C > T	p.Pro 3082Ser	-	+0.67	+0.67	+0.67	Severe	Yes	Autism. Hyperactivity, aggressiveness	slight telelia, ligamentous hyperlaxity	alteration of the posterior periventricular signal, low-signal stria in the supratrigonal area, modest ectasia of the regional perivascular spaces.

**Abbreviations**: H.: height. W.: weight. H.C.: head circumference. N.R.: not reported. I.D.: intellectual disability. L.D.: language delay. M.D.: motor delay. PKU: phenylketonuria; RRI: recurrent respiratory infections. *: International nomenclature of mutations

**Table 2:** Facial dysmorphisms and other physical characteristics of affected individuals with KMT2C mutation.

Features	Kleefstra et al. [12] (2012)	Koemans et al. (2017) [13]					Faundes et al. [9] (2018)			Schoch et al. [14] (2020)	Our Case
Sex	F	M	M	M	F	F	F	F	F	F	F
Age	15 Years	29 Years	31 Years	15 Years	7 Years	10 Years	17 Years	4 Years	5 Years	6 Years	6 Years
Microcephaly	+	−	−	−	+	+	+	+	−	−	−
Macrocephaly	−	−	−	−	−	−	−	−	−	+	+
Brachycephaly	+	−	−	−	−	−	−	−	−	−	−
Plagiocephaly	−	−	−	−	−	+	−	+	−	+	−
Coarse facies	+	−	−	−	−	−	−	−	−	−	−
Broad and rounded forehead	−	−	−	−	−	−	−	−	−	+	+
Deep set eyes	−	−	−	−	−	−	−	−	−	+	−
Marked infra-orbital creases	−	−	−	−	−	−	+	−	−	−	−
Midface hypoplasia	+	−	−	−	−	−	−	−	−	−	−
Flattened midface	−	+	−	−	+	−	−	−	−	−	−
Hypertelorism	+	−	−	−	−	−	−	−	−	−	+
Down-slanting palpebral fissures	−	−	−	−	−	−	+	+	−	−	−
Ptosis							+				
Synophrys	+	−	−	−	−	−	−	−	−	−	−
Arched eyebrows	−		−	−	−	−	−	+	−	−	−
Prominent eyebrows	−	+	−	+	−	−	−	−	−	−	−
Short nose	−	−	−	−	−	−	−	+	−	+	−
Nose with saddle bridge and bulbous tip	−	−	−	−	−	−	−	−	−	−	+
Narrow philtrum	−	−	−	−	−	−	−	−	−	+	−
Tented and cupid-bowed upper lip	+	−	−	−	−	−	−	−	−	−	−
Thick and everted lower lip	+	−	−	−	+	−	−	−	−	−	−
Thin upper lip, down-turned mouth, and misaligned teeth	−	−	−	−	−	−	−	−	−	+	−
High palate							+				
Pointed chin	+	−	−	−	−	−	−	−	−	−	−
Dysplastic ear helices	+	+	−	+	−	−	−	−	−	−	−
Preauricular tag	−	−	−	−	−	−	+	−	−	−	−
Duplicated right Thumb and left	−	−	−	−	−	−	+	−	−	−	−
Hearing loss (sensorineural)	−	−	−	−	−	−	+	−	−	−	−
Scoliosis	−	−	+	−	−	−	−	−	−	−	−
Thoracal kyphosis	−	+	−	−	−	+	−	−	−	−	−

## Data Availability

Not applicable.

## Supplementary Materials

The following supporting information can be downloaded at: https://www.mdpi.com/article/10.3390/pediatric14010019/s1, Figure S1. Flowchart of literature search results.
