# Supplementary figures and images for: De Novo Mutation in KMT2C Manifesting as Kleefstra Syndrome 2: Case Report and Literature Review
Source: Pediatr Rep. 2022 Mar 11;14(1):131–9. doi: 10.3390/pediatric14010019 (PMC8954887; doi:10.3390/pediatric14010019)

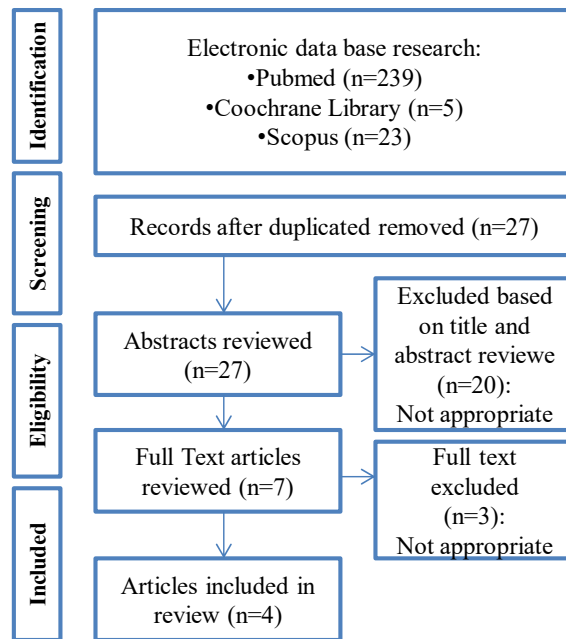

**Figure S1.** Flowchart of literature search results (Supplementary file on line only).

Supplement: Supplementary file 1 [file pediatrrep-14-00019-s001.zip › pediatrrep-1556696-supplementary.pdf]
